# The Characteristics, Prevalence, and Risk Factors of Drug-Induced Liver Injury Among Brucellosis Inpatients in Xinjiang, China

**DOI:** 10.3389/fphar.2021.657805

**Published:** 2021-05-10

**Authors:** Maermaer Tuohutaerbieke, Xinjie Li, Yue Yin, Wei Chen, Dongmei Wu, Zhize Mao, Jiamixi Mamuerjiang, Yimin Mao, Tao Shen

**Affiliations:** ^1^ Department of Microbiology and Infectious Disease Center, School of Basic Medical Sciences, Peking University, Beijing, China; ^2^ Department of Infectious Diseases, Shawan County People’s Hospital, Xinjiang, China; ^3^ Division of Gastroenterology and Hepatology, Shanghai Institute of Digestive Disease, Renji Hospital, Clinical Research Center, Shanghai Jiao Tong University School of Medicine, Shanghai, China

**Keywords:** rifampicin, doxycycline, RUCAM, epidemiology, drug-induced liver injury

## Abstract

**Background:** We investigated the prevalence, demographic and clinical features, and risk factors associated with drug-induced liver injury (DILI) during the treatment of brucellosis inpatients in a retrospective study.

**Methods:** We collected the clinical data of 782 brucellosis inpatients admitted at the Shawan County People’s Hospital, Xinjiang, from 2015–2019. All cases were re-evaluated using the international consensus of DILI criteria and RUCAM rating scale. 71 patients were confirmed as DILI cases and compared with 523 other patients with normal liver function.

**Results:** It was indicated that DILI occurred with a prevalence of about 9.08% among brucellosis inpatients receiving drug therapy. Hepatocellular injury was the most common type of DILI (61.97%, 95% confidence interval [CI] 50.34–72.37), followed by mixed (23.94%, 95% CI 15.52–35.04) and cholestatic types (14.08%, 95% CI 7.83–24.02). In addition, 13.64% of the hepatocellular DILI cases fulfilled Hy’s law criteria and only two cases (2.82%) progressed to severe DILI. Most patients adopted the combination of rifampicin, antipyretic analgesics, anti-infective agents, and traditional Chinese medicine for the treatment of brucellosis, with all the 71 patients taking rifampicin as the drug of choice. Multivariable logistic regression analyses indicated that obesity, regular alcohol intake, and decreased serum albumin were the independent risk factors of DILI in patients with brucellosis after adjusting for gender, age, and ethnicity.

**Conclusion:** DILI occurred in a minority of inpatients diagnosed with brucellosis receiving rifampicin-based therapeutic regimen. In addition, obesity, alcohol abuse, and decreased serum albumin were valuable predictors of the risk of DILI in patients with brucellosis.

## Introduction

Drug-induced liver injury refers to liver damage induced by various prescription or non-prescription chemical drugs, biological agents, traditional Chinese medicines, dietary supplements, and their metabolites, which is one of the most frequent and serious adverse drug reactions as well as the leading challenge in clinical treatment (Navarro and Senior, 2006).

Brucellosis, one of the most common zoonotic infectious diseases in the world, is a transmissible infectious disease caused by the invasion of *Brucella burgdorferi*. A previous study reported that it accounts for more than 500,000 new human infections each year in over 170 countries and regions worldwide, especially in the developing countries (Pappas et al., 2006). The annual incidence can reach up to 1/1000 in brucellosis endemic areas such as Latin America and the Middle East (Buzgan et al., 2010). In China, the rapid development of animal husbandry and tourism in the past few years has raised the risk of human infection, with 44,036 cases of human infection reported in 2019 alone, and an annual incidence rate of 3.25 infections per 100,000 people. From 2015–2019, the incidence of brucellosis in China fluctuated between 2.73–4.18 per 100,000 people, and was significantly higher in pastoral areas such as Xinjiang, Inner Mongolia, Gansu, and Qinghai (Jiang et al., 2020).

Pathogen transmissions for brucellosis mainly occur via inhalation, skin abrasion, consumption of contaminated food, and contact with mucous membranes. Individual infections can be attributed to contact with infected animals and their excrement, or by eating contaminated meat or dairy products. Brucella, an intracellular bacterium, readily escapes the therapeutic of antibiotics and restrains immune response, thereby leading to recurrent diseases which are difficult to effect a radical cure. Thus, the real incidence of brucellosis could be 10–25 times higher than the reported incidence (Avijgan et al., 2019).

Previous studies have reported that brucella are responsible for damaging either the nervous system, or the gastrointestinal tract, hepatobiliary, genitourinary, musculoskeletal, cardiovascular, and dermatological systems (Solera et al., 1997). The liver, which serves as an important defense against brucella infection, has also shown varying degrees of involvement in human brucellosis including the elevation of transaminase levels, enlargement of the liver and spleen, chronic suppurative disease, and acute hepatitis (Ozturk-Engin et al., 2014). Combination of rifampin with other drugs is recommended for the treatment of brucellosis in the clinical practice. However, been considered as one of the common causes of DILI, rifampin has led to great concern in anti-tuberculosis therapy with frequent reported of toxicity, and possibly adverse outcomes (Centers for Disease Control and Prevention, 2001a; Centers for Disease Control and Prevention, 2001b). Moreover, the digestive aids and anti-inflammatory drugs constantly used to treat brucellosis including herbal and antibiotic abuse, often lead to liver injury during the clinical treatment of brucellosis. Despite brucella intrinsically triggering abnormal liver function, liver injury caused by brucellosis-related therapeutic drugs has not yet raised sufficient attention. Therefore, it is vital to uncover the characteristics and causes of DILI in patients diagnosed with brucellosis and recommend interventions for promoting therapeutic efficacy.

Herein, we conducted a retrospective study to investigate inpatients hospitalized with brucellosis in Shawan County People’s Hospital, Xinjiang region, China from 2015–2019. The study analyzed the clinical characteristics, prevalence, and risk factors which contribute to DILI associated with brucellosis treatment. To some extent, the results would provide clinical guidance for the effective control of the liver injury process caused by brucellosis treatment.

## Materials and Methods

### Data Collection

We retrospectively collected the clinical data of 782 brucellosis patients hospitalized in Department of Infectious Diseases, Shawan County People’s Hospital of Xinjiang province from January 2015 to December 2019. The Xinjiang autonomous region is one of the five major pastoral areas in China, whose incidence of brucellosis ranks first in China. Shawan County is in the northwest of Xinjiang and is also one of the three national surveillance sites for brucellosis in China **(** Supplementary Figure S1 **)**. Shawan County People’s Hospital is the designated hospital for brucellosis treatment. Residents in surrounding areas with a history of pastoral exposure and suspicious symptoms such as fever, fatigue, and musculoskeletal pains would come for brucellosis diagnosis in the first instance. In the present study, the following data was collected for all enrolled patients: 1) Demographics; 2) Disease history and alcohol consumption history; 3) Information of the implicated drugs which may have caused the DILI including the types of implicated drugs and multi-drugs therapeutic regimen. In addition, the time of onset after starting the drug and the time of recovery after stopping the drug was also recorded; 4) Symptoms and signs including time of occurrence, time of disappearance, and symptoms during hospitalization were recorded in detail; 5) Serum biochemical indexes such as ALT, AST, ALP, TBil, DBil, prothrombin time (PT), and international normalized ratio (INR) before and during the DILI event; 6) Etiology analysis of other causes which may have resulted in liver damage (including hepatitis A virus (HAV), hepatitis B virus (HBV), hepatitis C virus (HCV), hepatitis E virus (HEV), herpes virus, Wilson’s disease, and autoimmune hepatitis); and 7) Severity of liver damage during the DILI event.

### Causality Assessment

**FIGURE 1:**
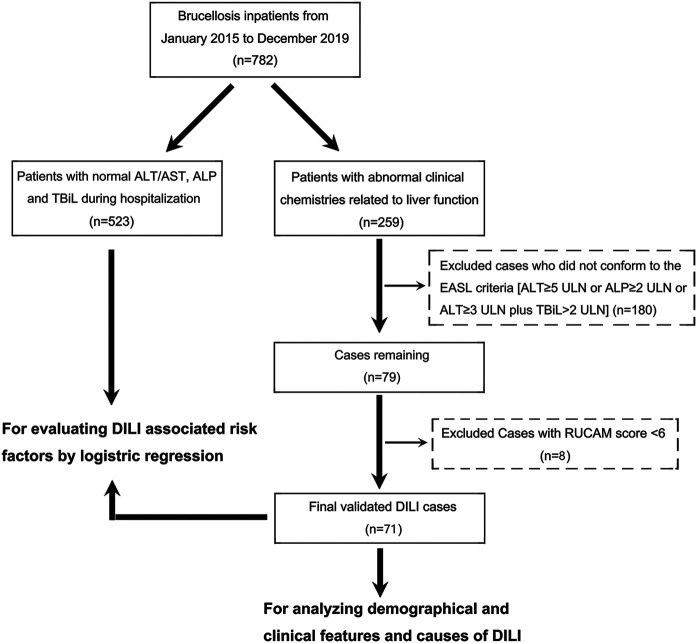
A flow diagram showing the recruitment of brucellosis-associated DILI patients in this study during a 5 year observation period at the Shawan County People’s Hospital (2015–2019).

The 782 brucellosis patients were then divided into two groups, 523 patients with normal liver function and 259 patients with abnormal liver function, according to the maximal levels of serum liver chemistries (ALT, AST, ALP, and TBil) during hospitalization. For the 259 patients with abnormal liver function, 180 were excluded because they did not conform to the European Association for the Study of the Liver (EASL) recommendation which includes alanine aminotransferase (ALT) ≥ 5 × upper limits of normal (ULN) or alkaline phosphatase (ALP) ≥ 2 × ULN or ALT ≥ 3 × ULN and total bilirubin (TBil) ≥ 2 × ULN (European Association for the Study of the Liver, 2019). Following this criterion, patients with mild drug-related biochemical abnormalities would be excluded from the diagnosis of DILI, which increases the reliability of the diagnosis to a certain extent. DILI causality assessment of the remaining 79 patients was reviewed and re-evaluated by local senior gastroenterologists according to the Roussel Uclaf Causality Assessment Method (RUCAM) (Yu et al., 2017). The patients with scores greater than or equal to 6 (“probable”, *n* = 71) were eventually included in our study (Figure 1).

### Clinical Presentation

The clinical types of DILI can be divided into three types based on the R value calculated using the following formula: R value = serum [ALT/ (ALT ULN)]/ [ALP/ (ALP ULN)]. The patients were classified as cholestatic if R value ≤2.0, mixed if R value ranged from 2.0 to 5.0, and hepatocellular if R value ≥5.0.

The classification of DILI severity was based on the international DILI Expert Working Group’s severity index, which includes four grades (mild, moderate, severe, and fatal/transplantation) (Aithal et al., 2011; European Association for the Study of the Liver, 2019). Mild level indicates DILI cases with ALT ≥5 or ALP ≥2 and TBL <2 ULN, while moderate level indicates ALT ≥5 or ALP ≥2 and TBL ≥2 ULN, or symptomatic hepatitis. On the other hand, severe DILI indicates ALT ≥5 or ALP ≥2 and TBL ≥2 ULN, or symptomatic hepatitis and one of the following criteria: (1) INR ≥1.5; (2) Ascites and/or encephalopathy, disease duration <26 weeks, and absence of underlying cirrhosis; (3) Other organ failures due to DILI. Finally, fatal/transplantation level indicates death or liver transplantation due to DILI.

### Diagnosis and Treatment

According to the “Chinese Guidelines for the Diagnosis and Treatment of Brucellosis” enacted by the Ministry of Health in 2012 (Ministry of Health of the People's Republic of China, 2012), patients with brucellosis are diagnosed by combining the epidemiological history, clinical manifestations, and laboratory tests: (1) Epidemiological history: patients have a history of close contact with confirmed animal brucellosis case, brucellosis-contaminated animal products, brucellosis cultures, or living in brucellosis endemic areas. (2) Clinical manifestations: patients suffer from fever, fatigue, sweats, myalgia, arthralgia or swelling of liver, spleen, lymph nodes, and testicles. (3) Standard tube agglutination test (SAT): the brucellosis total antibody titer is greater than or equal to 1:100++ or titers reach 1:50++ for patients with a duration of more than one year; or titers exceeds or equals 1:100++ for patients with a history of brucellosis vaccination within six months. (4) Complement binding test (CFT): antibody titer is greater than or equal to 1:10++. (5) Brucellosis anti-human immunoglobulin test (Coomb’s): antibody titer is higher than or equal to 1:400++. The diagnosis can be confirmed if both (1) and (2) are present and any one of (3), (4), and (5) is presented.

Treatment for human brucellosis strictly followed the “Chinese Guidelines for the Diagnosis and Treatment of Brucellosis” (Ministry of Health of the People's Republic of China, 2012), which is overall consistent with the Brucellosis Reference Guide: Exposures, Testing, and Prevention updated by the United States CDC (Al-Tawfiq, 2008; Centers for Disease Control and Prevention and National Center for Emerging and Zoonotic Infectious Diseases, 2017). The brucellosis treatment options are summarized as follows: (1) The principle of treatment is early, combined, sufficient, full course of treatment, and to extend the course of treatment to prevent recurrence and chronic symptoms if necessary. (2) Rifamycin and tetracycline are commonly used, and quinolones, sulfonamides, aminoglycosides and third generation cephalosporins can also be used. Pay attention to monitoring blood routine, liver, and kidney function during treatment. (3) For acute cases, doxycycline combined with rifampicin or streptomycin are used as the first-line regimen. In instances where the patients cannot use first-line drugs or have poor results, the following options can be chosen: doxycycline combined with compound sinomine or tobramycin; rifampicin combined with fluoroquinolones. (4) Fluoroquinolones or third-generation cephalosporins may be added to refractory cases. (5) For chronic acute attack cases, the usage was same as that for acute phase and some cases need 2-3 courses of treatment.

### Statistics

All the data were analyzed and graphs plotted using Graphpad Prism 7.0, with the exception of data having special instructions. Continuous variables were presented as median and IQR (inter quartile range). Comparisons between groups were done using the Mann-Whitney U test for the data that did not conform to a normal distribution. Categorical variables were expressed as percentages, and comparisons between groups were made using the χ^2^ test. In addition, single variable and multivariable logistic regression models were established to explore the risk factors associated with DILI in brucellosis patients. The variables with *p* < 0.2 after conducting univariate logistic regression were further included in the multifactor stepwise regression analysis, and models corrected for demographic factors such as patients’ age, gender, and ethnicity were provided. Relative risk was estimated by calculating odds ratio (ORs) and adjusted odds ratios (aORs) with corresponding 95% confidence intervals (CIs). A two-tailed *p* < 0.05 was statistically significant and the risk factor analysis was performed using SPSS 26.0.

## Results

### The Prevalence of DILI Among the Brucellosis Patients

The total number of brucellosis patients hospitalized in the Shawan County People’s Hospital from January 2015 to December 2019 was 782. The liver function of 71 patients among the 259 patients with abnormal liver function met both the EASL serological criteria for DILI identification and RUCAM scoring (≥6) (Figure 1). Moreover, the probability of developing DILI among patients with brucellosis during the 5 years observation period was 9.08% (95% CI: 7.26–11.30) (Table 1). This means that DILI presented frequently among the brucellosis patients receiving clinical treatment in Xinjiang region.

**TABLE 1:** Evaluation of the proportion of DILI cases among brucellosis inpatients in Shawan County of Xinjiang province from January 2015 to December 2019.

Years	Number of brucellosis inpatients	Number of inpatients with abnormal liver function	Number of DILI inpatients	Proportion of DILI (%)^a^	95% CI
2015	214	53	26	12.15	[8.43–17.21]
2016	80	22	12	15.00	[8.79–24.41]
2017	212	88	12	5.66	[3.27–9.63]
2018	149	59	16	10.74	[6.72–16.73]
2019	127	37	5	3.94	[1.69–8.89]
Total	782	259	71	9.08	[7.26–11.30]

a The proportion of DILI = annual number of DILI in inpatients in this hospital/ annual number of brucellosis patients.

### Demographic Characteristics of Brucellosis-Associated DILI Patients

In this study, 71 cases were finally identified as having occurrence of DILI, with the number of males (55, 77.46%) being significantly higher than the number of females (16, 22.54%) (Table 2). The age of patients ranged from 4–74 years old, with the highest proportion 52.11% (*n* = 37) of patients being between 40–59 years, followed by 18–39 years (22, 30.99%). It is worth noting that only a few patients were older than 60 or younger than 18 years (Table 2). However, there were no significant differences in the proportion of patients with DILI by sex, age, or ethnicity in the corresponding groups of patients with brucellosis (Supplementary Figure S2). Majority of the patients with DILI in brucellosis were middle-aged males, which can be attributed to the greater access to livestock production and increased opportunities for infection through contact with sick animals and handling of animal effluents. This data indicates that the tendencies of age, sex and ethnicity in DILI occurrence greatly contributed to the differences in the composition of brucellosis patients.

**TABLE 2:** Demographic and clinical features of 71 brucellosis-associated DILI hospitalized in Shawan County People’s Hospital from January 2015 to December 2019.

	Number	%	95% CI
Gender			
Male	55	77.46	[66.48–85.63]
Female	16	22.54	[14.37–33.52]
Age (year)			
≥ 60	7	9.86	[4.86–18.98]
40–59	37	52.11	[40.69–63.32]
18–39	22	30.99	[21.44–42.48]
<18	5	7.04	[3.05–15.45]
Ethnicity			
Han	20	28.17	[19.04–39.54]
Non-Han	51	71.83	[60.46–80.96]
Latent period			
≤ 7 days	8	11.27	[5.82–20.69]
> 7 days and ≤ 14 days	63	88.73	[79.31–94.18]
ALT (U/L)			
≥ 5 × ULN	57	80.28	[69.58–87.87]
≥ 3 × ULN and < 5 × ULN	8	11.27	[5.82–20.69]
< 3 × ULN	6	8.45	[3.93–17.24]
AST (U/L)			
≥ 5 × ULN	37	52.11	[40.69–63.32]
≥ 3 × ULN and < 5 × ULN	23	32.39	[22.66–43.94]
< 3 × ULN	11	15.49	[8.88–25.65]
ALP (U/L)			
≥ 2 × ULN	17	23.94	[15.52–35.04]
< 2 × ULN	54	76.06	[64.96–84.48]
TBil (μmol/L)			
≥ 5 × ULN	1	1.41	[0.07–7.56]
≥2 × ULN and < 5 × ULN	6	8.45	[3.93–17.24]
<2 × ULN	64	90.14	[81.02–95.14]
Clinical types			
Hepatocellular injury (R ≥ 5)	44	61.97	[50.34–72.37]
Conform to Hy’s law	6	13.64	[6.40–26.71]
Others	38	86.36	[73.29–93.60]
Cholestatic injury (R ≤ 2)	10	14.08	[7.83–24.02]
Mixed injury (2 < R < 5)	17	23.94	[15.52–35.04]
Severity classification			
Mild	64	90.14	[81.02–95.14]
Moderate	5	7.04	[3.05–15.45]
Severe	2	2.82	[0.50–9.70]
Fatal/transplantation	0	0	[0–5.13]

ALT, alanine aminotransferase; ALP, alkaline phosphatase; AST, aspartate aminotransferase; TBil, total bilirubin.

Patients with underlying liver disease (3 patients with chronic hepatitis C and 4 with chronic hepatitis B) were not excluded from this study to better reflect the real situation of DILI in patients with brucellosis, and all patients with viral hepatitis had a history of antiviral treatment. A comparison of the liver function parameters such as ALT, AST, ALP, and TBil before and after receiving brucellosis treatment indicated that the serological markers of all DILI cases increased sharply after the patients received brucellosis-related treatment (*p* < 0.0001)**,** either for patients with hepatocellular or cholestatic or mixed injuries **(** Supplementary Figure S3 **)**. Further basic information on the 71 patients with brucellosis-related DILI including their clinical symptoms, previous diseases history and laboratory tests results were detailed in Supplementary Tables S1, S2.

### Clinical Manifestations of Patients With Brucellosis-Related DILI

The serum liver function indexes were recorded when abnormal liver function was observed in all 71 patients for the first time after receiving the brucellosis-related treatment. The obtained results indicated that 57 patients (95% CI 69.58–87.87) had serum ALT ≥5 × ULN, eight patients (95% CI 5.82–20.69) had serum ALT ≥3 × ULN and <5 × ULN, and six patients (95% CI 3.93–17.24) presented serum ALT <3 × ULN, respectively. Majority of the patients had hepatocellular injury type (61.97%, 95% CI 50.34–72.37), followed by mixed type (23.94%, 95% CI 15.52–35.04) and cholestasis type (14.08%, 95% CI 7.83–24.02). In addition, 13.64% of the hepatocellular DILI cases fulfilled Hy’s law criteria and only two cases (2.82%) progressed to severe DILI (Table 2).

With the exception of two patients who developed severe DILI, most of the patients had mild or moderate symptoms of liver injury, which merely showed changes in the serum biochemical parameters. Furthermore, no obvious clinical jaundice and ascites were observed, and no fatal DILI cases were found in our study (Table 2).

In addition, we found that four of the 71 DILI patients were re-hospitalized due to the recurrence of brucellosis half a year to a year after initial hospitalization. However, the latency after treatment with the similar regimen (containing rifampicin and doxycycline) was significantly shortened from 8-12 days of the first hospitalization to 4–5 days of the second hospitalization. Moreover, the characteristics of liver injury in the second hospitalization were similar to the first hospitalization (Supplementary Table S3).

### Characteristics of Changes of Liver Biochemistries

**FIGURE 2:**
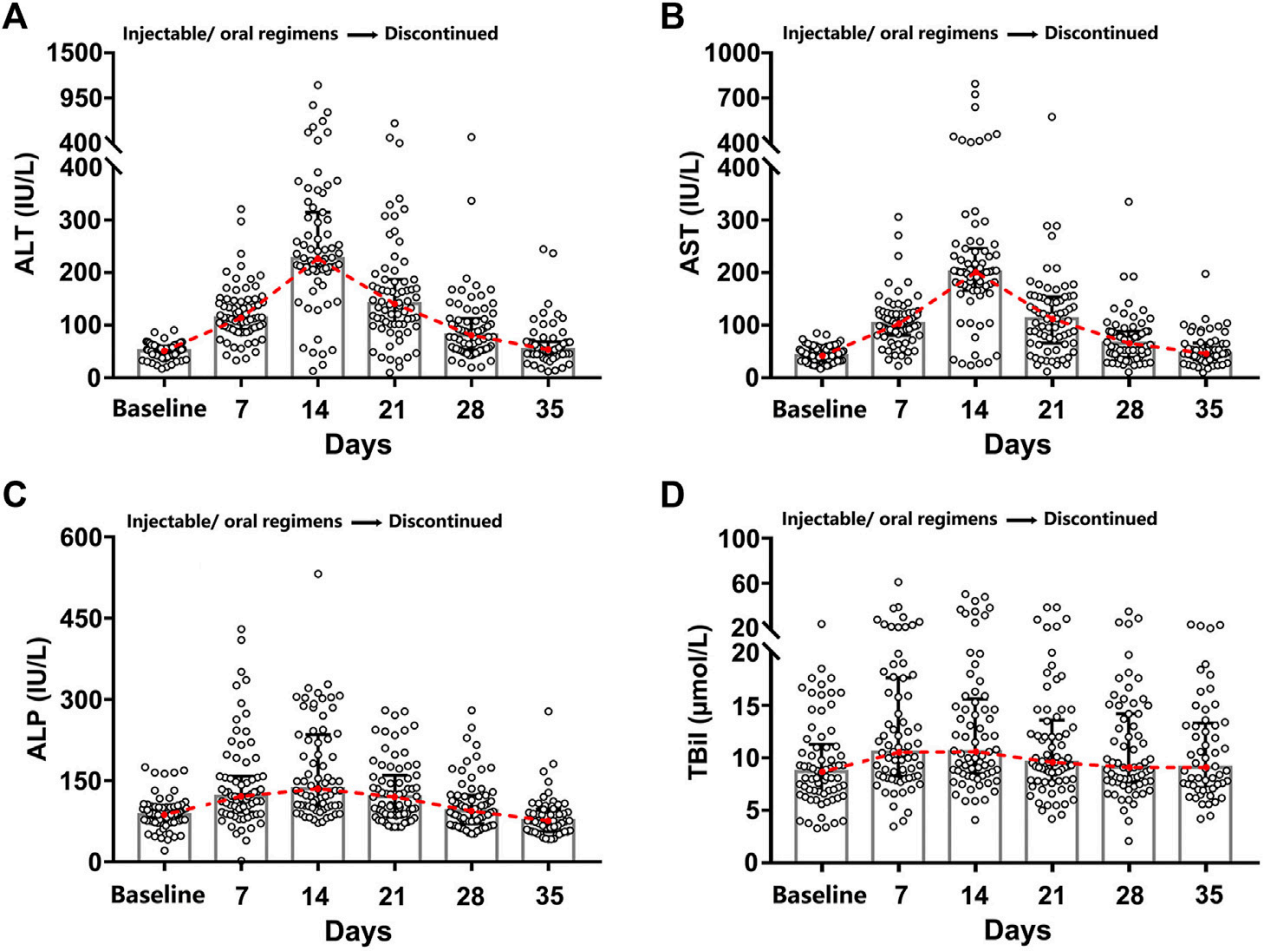
Dynamic hepatic function indexes diagram. The treatment (injectable/oral regimens) was immediately discontinued for one to three weeks once abnormal liver function appeared. Four main hepatic function indexes (ALT **(A)**, AST **(B)**, ALP **(C)**, and TBil **(D)**) were recorded for the 71 DILI patients before and after brucellosis-specific treatment.

All the patients enrolled in this study had acute DILI, which was characterized by the appearance of an abnormal liver function (ALT, AST, ALP, and TBil) within seven to 14 days of treatment with injectable/oral regimens and getting back to the pre-treatment baseline levels within one to three weeks after discontinuation of the medications (Figure 2). Furthermore, the analysis of various age groups indicated that adolescents and elderly people exhibited milder symptoms of liver damage than young adults (TBil, *p* = 0.008). However, no significant differences were found between males and females with regards to the extent of liver damage (Supplementary Figure S4).

### Implicated Drugs in DILI Among Patients With Brucellosis

**FIGURE 3:**
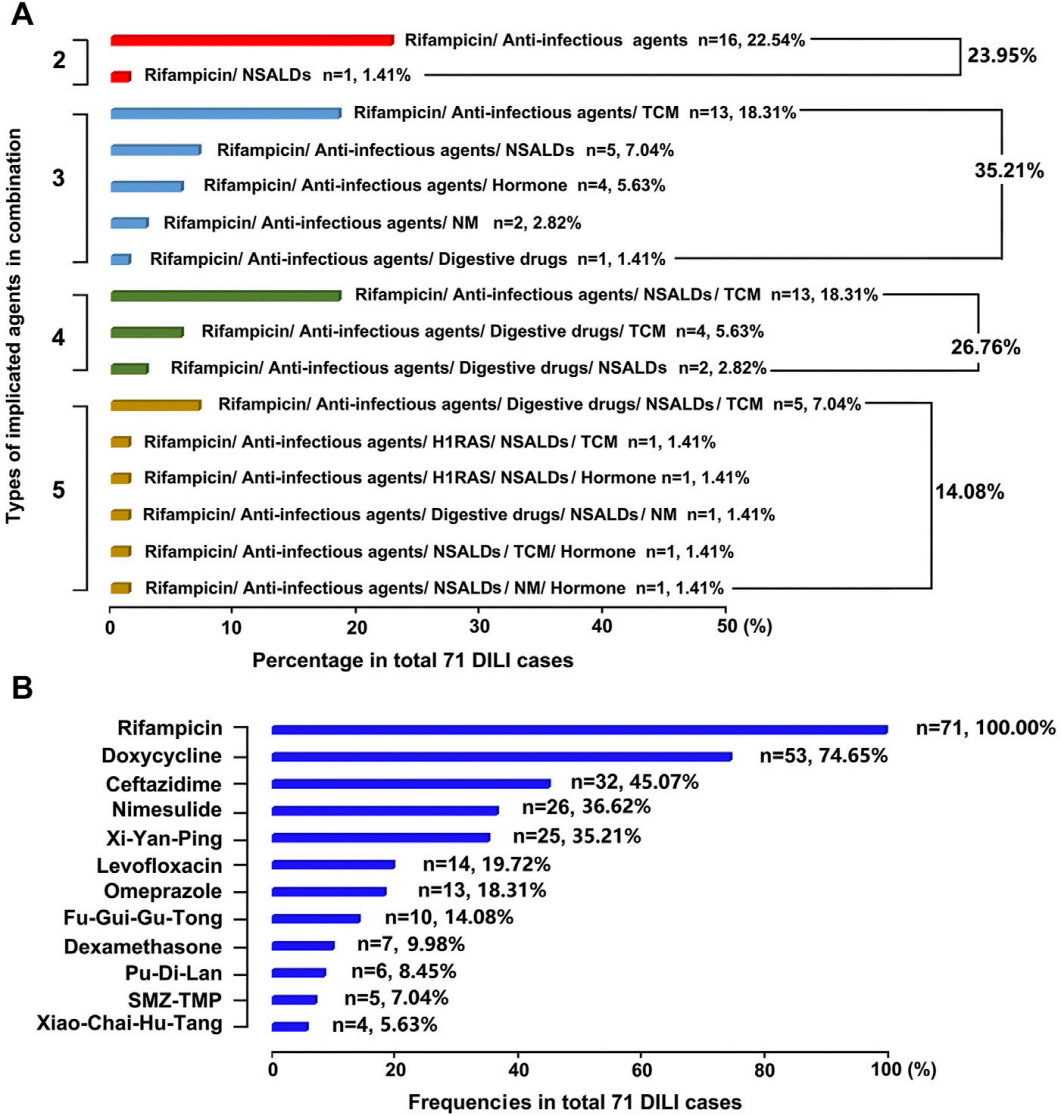
Causes of DILI in this study **(A)** Implicated DILI drugs were displayed according to their types in combination **(B)** Frequencies of implicated specific DILI drugs were ranked in the total 71 DILI cases.

There were mainly eight classes of drugs among the therapeutic regimens of the 71 brucellosis-related DILI patients: rifampicin, anti-infectious agents, nonsteroidal anti-inflammatory drugs (NSALDs), H1 receptor antagonists (H1RAS), digestive drugs, hormones, traditional Chinese medicine (TCM), and natural medicine (NM) (Supplementary Table S4). Each patient received at least two classes of drug combination therapy, and three or four classes of drug combination was the most common regimen (35.21% and 26.76% of patients, respectively), followed by two classes of drug combination regimens. In addition, five classes of drug combination regimens were the least common, with only 14.08% of patients opting for this schedule. The combination of rifampicin and anti-infectious agents was the most common regimen because it was chosen by 16 patients (22.54%) (Figure 3A).

There were 23 specific drugs involved in this cohort, and the top 10, in descending order of frequency of use, were: rifampicin (71, 100%), doxycycline (53, 74.65%), ceftazidime (32, 45.07%), nimesulide (26, 36.62%), Xi-Yan-Ping (25, 35.21%), levofloxacin (14, 19.72%), omeprazole (13, 18.31%), Fu-Gui-Gu-Tong (10, 14.08%), dexamethasone (7, 9.98%), and Pu-Di-Lan (6, 8.45%) (Figure 3B).

### Independent Risk Factors for DILI in Brucellosis Patients

The results obtained after conducting single variable logistic regression analysis indicated that patients with obesity (OR = 57.915, 95% CI: 7.138–469.937, *p* < 0.0001) or had a history of regular alcohol consumption (OR = 3.844, 95% CI: 1.254–11.782, *p* = 0.018) had a higher incidence of DILI. In addition, fever (OR = 1.670, 95% CI: 0.997–2.798, *p* = 0.051) and decreased serum albumin (OR = 0.933, 95% CI: 0.883–0.986, *p* = 0.014) were also associated with DILI. It is worth noting that the risk of DILI did not differ significantly by age, sex, and ethnicity **(** Supplementary Table S5 **).** We then included the complete data of 594 patients (including 523 patients with normal liver function and 71 patients with DILI) in the multivariable logistic stepwise regression model. The obtained results indicated that fever, obesity, frequent alcohol consumption, and decreased serum albumin were independent risk factors for the development of DILI in patients with brucellosis. Moreover, regular alcohol consumption (OR = 3.893, 95% CI: 1.180–12.840, *p* = 0.026), decreased serum albumin (OR = 0.915, 95% CI: 0.860–0.973, *p* = 0.005), and obesity (OR = 66.144, 95% CI: 7.956–549.921, *p* < 0.0001) were still significantly associated with DILI symptoms in patients with brucellosis when the model was adjusted for age, sex, and ethnicity, which could in some extent predict the risk of DILI in patients with brucellosis (Table 3).

**TABLE 3:** Multivariable logistic regression model for risk factors of DILI among brucellosis inpatients

Variables	Crude Odds ratio (95%CI)	*P* Value	Adjusted Odds ratio^a^ (95%CI)	*P* Value
Fever, > 37.5°C (yes vs. no)	1.590 (0.922–2.743)	0.095	1.561 (0.896–2.720)	0.116
Obesity^b^ (yes vs. no)	63.630 (7.731–523.715)	<0.0001	66.144 (7.956–549.921)	<0.0001
Regular alcohol intake (yes vs. no)	4.070 (1.246–13.295)	0.020	3.893 (1.180–12.840)	0.026
Serum albumin, g/L	0.935 (0.882–0.990)	0.022	0.915 (0.860–0.973)	0.005

a Adjusted for age, gender, and ethnicities.

b BMI is greater than 30 for obesity.

## Discussions

This study focused on the patients hospitalized with brucellosis and suffered from DILI in the Infectious Diseases Department of Shawan County People’s Hospital from 2015–2019. The study analyzed the data of liver function parameters, with an overarching goal of uncovering the characteristics and risk factors of DILI associated with brucellosis. Previous studies have reported that the failure and recurrence rates after brucellosis treatment have been rising over the past 20 years, which has increased from 4.6%–24% for oral regimens and from 5%–8% for oral/injectable regimens (Falagas and Bliziotis, 2006; Roushan et al., 2006). The fact that brucella inherently affects liver function, coupled with liver damage caused by rifampicin-based drug therapy that further increases the burden of liver function, contributes to the increased failure rate of brucellosis treatment. This study enrolled patients who met the ESAL criteria for liver injury, and all cases of abnormal liver function that were not consistent with the serological criteria were excluded from the final DILI cases. Nevertheless, DILI was present in about 9% of the patients hospitalized with brucellosis. DILI associated with brucellosis disease, mostly acute, demonstrated dynamic changes in the liver function. However, the liver could gradually regain normal function after two to three weeks of suspending the drug therapy, where the liver injury displayed a downward trend.

Patients in this cohort used rifampicin, an anti-tuberculosis drug, as the main drug type for brucellosis-related DILI, which is consistent with the main drug type causing DILI in mainland China (Shen et al., 2019). However, the brucellosis treatment-associated liver injury also requires comprehensive consideration of the effects and interactions of different drugs because single-drug regimens have been obsoleted, and thus the combined use of multiple drugs and abuse of antibiotics and herbs further increases the incidence of DILI. Such as the second most used drug, Doxycycline, was also reported to cause drug-induced liver injury (Bjornsson et al., 2013). In addition, the licensed dose of rifampin recommended for brucellosis treatment (200mg doxycycline plus 600–900 mg rifampin for at least six weeks) is also higher than that of the anti-tuberculosis treatment (10 mg/kg body weight, 2–3 times a week, up to a maximum of 600 mg each time), which may also lead to serious side effects (Pappas et al., 2005).

Comparisons were also performed between patients with normal liver function and those who developed DILI in order to explore the key risk factors triggering DILI in patients suffering from brucellosis. Previous studies have generally considered elderly people (age >60 years) to be a risk factor for the development of DILI because they possess a declining physiological function, diminished ability to bio-transform and excrete drugs, reduced tolerance, and increased sensitivity to drugs (Saukkonen et al., 2006; Abboud and Kaplowitz, 2007). In addition, females are thought to be more susceptible to drug-induced hepatotoxicity than males (Devarbhavi, 2011; Bjornsson et al., 2013). However, the results obtained in this study indicated that there were no significant differences in the risk of DILI among patients with varying age, gender, and ethnicities. The key risk factors predisposing DILI with brucellosis were obesity, regular alcohol consumption, and decreased serum albumin. A previous study reported that patients with malnutrition had reduced serum albumin concentrations, and given that many drugs in the serum require binding to high levels of protein, low protein-drug binding may result in greater distribution of free drugs to target organs and subsequent tissue toxicity (Greenblatt and Koch-Weser, 1974). Moreover, alcohol abuse acts as a key factor in the susceptibility of DILI in brucellosis patients, which is similar with tuberculosis patients (Dakhoul et al., 2018). Long-term alcoholism can lead to the degeneration of liver cells, with the necrosis and fibrosis resulting in a decrease of the liver metabolism and detoxification capacity. This further causes accumulation of drugs as well as their reactive metabolites in the liver, thereby leading to hepatic damage (Chen et al., 2016). Another study conducted on the Chinese people indicated that the risk of DILI in patients with a history of alcohol consumption is about 4.5 times higher than that of patients who do not drink alcohol (Nie et al., 2017). Obesity is another important risk factor in the formation of DILI. On one hand, the increased activity of cytochrome P450 (CYPs) (CYP2C9, CYP1A2, CYP2E1, and CYP2D6) in individuals with obesity could augment generation of toxic metabolites (Brill et al., 2012). On the other hand, the high body fat in obese individuals is associated with decreased drug clearance and the subsequent slower elimination of drugs and higher drug levels in the plasma (Chen et al., 2015).

In the treatment of brucellosis, monotherapy has been reported to be associated with a higher risk of failure compared to combination therapy (Skalsky et al., 2008). Given the necessity of combinatory medication in clinical treatment, it’s hard to assess or clarify the association between single agent and liver injury. As a pastoral area, Xinjiang is a vast territory with an uneven incidence of brucellosis. However, there could be plenty of patients infected with brucella who haven’t been hospitalized, who may have taken self-medication or outpatient medication and suffered from DILI. Unfortunately, we were unable to obtain clinical information and medication for these individuals, and we could only offer the prevalence of brucellosis-related DILI among hospitalized patients.

Overall, in consideration of the prevalence of DILI in brucellosis, a comprehensive examination of the liver function of the patient should be conducted prior to the treatment of DILI, and a personalized treatment plan should be formulated according to the basic liver function status of the patient. Treatment options can be substituted with rifampicin non-dependent regimens for patients prone to DILI, whereas caution should be taken while using herbs to reduce the incidence of DILI.

## Conclusion

To the best of our limited current knowledge, this research reported, for the first time, on the DILI cases among patients with brucellosis, and provided valid information on the prevalence, characteristics, and underlying risk factors. The obtained results could be indicative for early triage of clinical cases, targeted treatments, and help in curbing the unnecessary detrimental effects.

## Data Availability

The raw data supporting the conclusion of this article will be made available by the authors, without undue reservation.

## Glossary

ALB: Albumin
ALP: alkaline phosphatase
ALT: alanine aminotransferase
aOR: adjusted odds ratios
AST: aspertate aminotransferase
BMI: body mass index
CFT: complement binding test
CI: confidence level
Cr: creatinine
CYP: cytochrome P450
DBil: direct bilirubin
DILI: drug-induced liver injury
EASL: European Association for the Study of the Liver
FIB: plasma fibrinogen
GLO: globulin
GGT: gamma-glutamyl transpeptidase
HAV: hepatitis A virus
HBV: hepatitis B virus
HCV: hepatitis C virus
HEV: hepatitis E virus
H1RAS: H1 receptor antagonists
IBil: indirect bilirubin
IQR: inter quartile range;
NM: natural medicine
NSALDs: nonsteroidal anti-inflammatory drugs
OR: odds ratio
RUCAM: Roussel Uclaf causality assessment method;
SAT: standard tube agglutination test
TBA: total bile acid
TBil: total bilirubin
TCM: traditional Chinese medicine
TP: total protein
ULN: upper limits of normal
