# Supplementary material for: The Characteristics, Prevalence, and Risk Factors of Drug-Induced Liver Injury Among Brucellosis Inpatients in Xinjiang, China
Source: Front Pharmacol. 2021 May 10;12:657805. doi: 10.3389/fphar.2021.657805 (PMC8141917; doi:10.3389/fphar.2021.657805)
Supplement: Supplementary file 1 [file datasheet1.doc]

**The Characteristics, Prevalence, and Risk Factors of** **Drug-Induced Liver Injury among Brucellosis Inpatients in Xinjiang, China**

**Supplementary Materials**

**Maermaer Tuohutaerbieke^1, †^, Xinjie Li^1, †^, Yue Yin^1^, Wei Chen^2^, Dongmei Wu^2^, Zhize Mao^2^, Jiamixi Mamuerjiang^2^, Yimin Mao^3, *^, Tao Shen^1, *^**

^1^ Department of Microbiology and Infectious Disease Center, School of Basic Medical Sciences, Peking University, Beijing 100191, China

^2^ Department of Infectious Diseases, Shawan County People's Hospital, Xinjiang 833000, China

^3^ Division of Gastroenterology and Hepatology, Shanghai Institute of Digestive Disease, Renji Hospital, School of Medicine, Shanghai Jiao Tong University, Shanghai, China. Clinical Research Center, Shanghai Jiao Tong University School of Medicine, Shanghai 200001, China

**^†^**These authors contributed equally to the manuscript.

*******Corresponding authors:**

Tao Shen, MD & PhD

Tel: 86-10-82805070; Fax: 86-10-82805136

Email: taoshen@hsc.pku.edu.cn

Yimin Mao, MD

Tel: +862158752345; Fax: +862163034707

Email: [maoym11968@163.com](mailto:maoym11968@163.com)

# 1 Supplementary Figures and Tables

- 1. **Supplementary Tables**

**Supplementary Table 1.** Clinical manifestations and previous medical histories of 71 Brucellosis-associated DILI patients at admission.

|  | **Number** | **Percentage（%）** | **95%CI** |
| --- | --- | --- | --- |
| **Manifestations** | | | |
| Fever | 49 | 69.01 | [57.52-78.56] |
| Sweating | 25 | 35.21 | [25.12-46.82] |
| Fatigue | 40 | 56.34 | [44.77-67.25] |
| Musculoskeletal pains | 50 | 70.42 | [58.98-79.77] |
| Dizziness/headache | 11 | 15.49 | [8.88-25.65] |
| Nausea/anorexia | 14 | 19.72 | [12.13-30.42] |
| Orchitis/Epididymitis | 6 | 8.45 | [3.93-17.24] |
| Ascites | 1 | 1.41 | [0.70-7.56] |
| Jaundice | 2 | 2.82 | [0.50-9.70] |
| Edema of lower limbs | 1 | 1.41 | [0.70-7.56] |
| **Previous medical histories** | | | |
| Viral hepatitis^a^ | 7 | 9.86 | [4.86-18.98] |
| Cholecystitis | 5 | 7.04 | [3.05-15.45] |
| Coronary heart disease | 1 | 1.41 | [0.70-7.56] |
| Diabetes | 3 | 4.23 | [1.15-11.7] |
| Obesity (BMI >30) | 9 | 12.68 | [6.81-22.37] |
| Electrolyte disorder | 3 | 4.23 | [1.15-11.7] |
| Carcinoma^b^ | 1 | 1.41 | [0.70-7.56] |
| Hemangioma | 2 | 2.82 | [0.50-9.70] |
| Mild anemia | 6 | 8.45 | [3.93-17.24] |
| Hypoproteinemia | 4 | 5.63 | [2.21-13.61] |
| Others^c^ | 7 | 9.86 | [4.86-18.98] |

^a^Viral hepatitis included 4 cases of positive HBsAg and 3 cases of positive anti-HCV, all of whom took medication regularly for control. ^b^Carcinoma included 1 case of gastric cancer. ^c^Others included 1 case of syphilis, 1 case of bronchopneumonia, 1 case of inactive pulmonary tuberculosis, 1 case of rheumatoid arthritis, 1 case of intervertebral disc herniation, 1 case of secondary thrombocytosis and 1 case of lacunar cerebral infarction.

**Supplementary Table 2. The maximal values of clinical chemistries in 71 cases with brucellosis-related DILI during hospitalization.**

| **ID** | **Gender** | **Age** | **Ethnicity** | **ALT**  **(IU/L)** | **AST**  **(IU/L)** | **GGT**  **(IU/L)** | **ALKP**  **(IU/L)** | **TBil**  **(μM)** | **DBil**  **(μM)** | **IBil**  **(μM)** |
| --- | --- | --- | --- | --- | --- | --- | --- | --- | --- | --- |
| 15139661 | male | 62 | Han | 237 | 232 | 28 | 97 | 447.9 | 257.9 | 190 |
| 15142660 | male | 58 | Han | 391 | 204 | 68 | 118 | 14.6 | 9 | 5.6 |
| 15143093 | male | 47 | Han | 144 | 113 | 33 | 76 | 38.9 | 22.5 | 16.4 |
| 19217240 | male | 73 | Kazakh | 97 | 95 | 239 | 326 | 38 | 21 | 17 |
| 15138896 | male | 52 | Han | 129 | 104 | 58 | 95 | 44.4 | 31 | 13.4 |
| 15140224 | male | 52 | Han | 367 | 246 | 135 | 146 | 18 | 10 | 8 |
| 15142777 | male | 55 | Hui | 236 | 182 | 165 | 129 | 19 | 12 | 7 |
| 15142854 | female | 57 | Han | 669 | 641 | 70 | 149 | 10.7 | 2 | 8.7 |
| 15143203 | male | 53 | Kazakh | 864 | 726 | 582 | 307 | 14.9 | 7.9 | 7 |
| 15144286 | male | 30 | Kazakh | 305 | 260 | 66 | 89 | 7 | 3.8 | 3.2 |
| 16157802 | male | 23 | Hui | 352 | 293 | 20 | 83 | 8.3 | 3.8 | 4.5 |
| 16158084 | male | 43 | Kazakh | 315 | 217 | 141 | 304 | 14.6 | 7.3 | 7.3 |
| 16160697 | male | 26 | Kazakh | 244 | 218 | 65 | 124 | 5.9 | 2.5 | 3.4 |
| 16170276 | male | 6 | Kazakh | 207 | 172 | 30 | 305 | 7.6 | 3.8 | 3.8 |
| 16171147 | female | 58 | Kazakh | 228 | 179 | 107 | 220 | 13.1 | 7.5 | 5.6 |
| 17175977 | male | 23 | Kazakh | 215 | 182 | 64 | 104 | 8.5 | 5.2 | 3.3 |
| 17178494 | female | 60 | Han | 202 | 190 | 39 | 103 | 9.5 | 5.4 | 4.1 |
| 17179136 | male | 40 | Kazakh | 223 | 203 | 70 | 198 | 8.9 | 5 | 3.9 |
| 17179245 | female | 58 | Han | 301 | 254 | 62 | 114 | 13.7 | 4.6 | 9.1 |
| 17179755 | male | 29 | Kazakh | 247 | 240 | 90 | 74 | 12.2 | 6.7 | 5.5 |
| 18192965 | female | 45 | Kazakh | 357 | 460 | 82 | 194 | 8.7 | 6 | 2.7 |
| 18193751 | female | 9 | Kazakh | 253 | 195 | 114 | 285 | 8.5 | 3 | 5.5 |
| 18194838 | male | 46 | Kazakh | 140 | 104 | 175 | 532 | 50.4 | 41 | 9.4 |
| 18194999 | male | 41 | Kazakh | 132 | 104 | 153 | 328 | 15.9 | 13.5 | 2.4 |
| 18196077 | male | 9 | Kazakh | 227 | 217 | 38 | 244 | 4.1 | 2 | 2.1 |
| 18196123 | male | 26 | Kazakh | 778 | 445 | 78 | 128 | 8.9 | 6.1 | 2.8 |
| 18196433 | male | 8 | Kazakh | 184 | 157 | 47 | 270 | 16.3 | 12.3 | 4 |
| 18196566 | male | 66 | Uygur | 202 | 177 | 69 | 135 | 14..4 | 8.3 | 6.1 |
| 18197175 | female | 36 | Kazakh | 206 | 298 | 42 | 149 | 10.6 | 6.4 | 4.2 |
| 18198330 | female | 63 | Hui | 203 | 311 | 73 | 149 | 47.9 | 40.5 | 7.4 |
| 18199152 | male | 33 | Kazakh | 375 | 206 | 127 | 156 | 12.1 | 7.7 | 4.4 |
| 18199475 | female | 31 | Han | 296 | 255 | 26 | 105 | 7.5 | 4.2 | 3.3 |
| 17178514 | female | 49 | Kazakh | 253 | 231 | 26 | 81 | 9.6 | 4.4 | 5.2 |
| 17179292 | male | 37 | Hui | 211 | 182 | 126 | 132 | 34.9 | 23.9 | 11 |
| 18196135 | male | 51 | Han | 211 | 167 | 85 | 108 | 9.3 | 4.8 | 4.5 |
| 18191934 | male | 42 | Hui | 221 | 161 | 111 | 177 | 7.7 | 5.9 | 1.8 |
| 17174598 | male | 37 | Han | 535 | 421 | 116 | 132 | 20 | 8.4 | 11.6 |
| 15145082 | male | 49 | Kazakh | 595 | 406 | 91 | 101 | 15.9 | 8.3 | 7.6 |
| 18205708 | female | 29 | Kazakh | 135 | 110 | 95 | 336 | 21.6 | 17 | 4.6 |
| 19212593 | male | 67 | Han | 211 | 166 | 92 | 302 | 6 | 4.2 | 1.8 |
| 17175270 | male | 37 | Han | 535 | 416 | 132 | 132 | 38.4 | 19.6 | 18.8 |
| 15145509 | male | 21 | Kazakh | 271 | 201 | 197 | 225 | 9.3 | 4.4 | 4.9 |
| 15142398 | male | 22 | Han | 206 | 209 | 315 | 195 | 14.6 | 8.6 | 6 |
| 17187289 | female | 41 | Kazakh | 58 | 32 | 34 | 410 | 12.7 | 5.3 | 7.4 |
| 16162715 | female | 41 | Kazakh | 152 | 120 | 247 | 274 | 19.9 | 11.4 | 8.5 |
| 17180857 | male | 48 | Hui | 13 | 27 | 15 | 303 | 17.3 | 12.4 | 4.9 |
| 18205953 | male | 30 | Kazakh | 25 | 30 | 164 | 292 | 13.7 | 6.4 | 7.3 |
| 19210674 | male | 66 | Han | 245 | 259 | 329 | 227 | 10.2 | 4 | 1.2 |
| 19216398 | male | 56 | Hui | 374 | 441 | 160 | 237 | 33.4 | 16.4 | 17 |
| 19210560 | male | 42 | Kazakh | 1108 | 796 | 99 | 104 | 18.9 | 5.6 | 13.3 |
| 16161113 | male | 44 | Kazakh | 67 | 43 | 119 | 293 | 15.8 | 9.2 | 6.6 |
| 16161927 | male | 57 | Kazakh | 241 | 146 | 89 | 141 | 9.6 | 6.2 | 3.4 |
| 15137369 | male | 48 | Kazakh | 213 | 179 | 115 | 123 | 31.6 | 17 | 14.6 |
| 15136891 | male | 44 | Han | 229 | 166 | 108 | 90 | 12 | 2 | 10 |
| 15142160 | male | 39 | Han | 213 | 202 | 87 | 92 | 10 | 7 | 3 |
| 15143141 | male | 35 | Kazakh | 209 | 183 | 121 | 105 | 9 | 7 | 2 |
| 15143295 | male | 36 | Kazakh | 264 | 204 | 129 | 162 | 9.5 | 7.6 | 1.9 |
| 15143594 | male | 47 | Kazakh | 236 | 198 | 76 | 83 | 8.8 | 6.7 | 2.1 |
| 15143911 | male | 43 | Han | 324 | 234 | 81 | 110 | 10 | 7.7 | 2.3 |
| 15135820 | male | 40 | Han | 211 | 183 | 95 | 104 | 7.4 | 6.5 | 0.9 |
| 15146704 | male | 48 | Kazakh | 216 | 192 | 49 | 78 | 8.3 | 6.4 | 1.9 |
| 15146408 | male | 45 | Hui | 361 | 241 | 78 | 100 | 12 | 7 | 5 |
| 15147544 | female | 52 | Kazakh | 222 | 201 | 52 | 88 | 9.1 | 7 | 2.1 |
| 15147728 | male | 35 | Kazakh | 435 | 317 | 81 | 154 | 11.5 | 6.9 | 4.6 |
| 15148027 | male | 28 | Kazakh | 216 | 175 | 73 | 89 | 10.4 | 7.2 | 3.2 |
| 15152608 | female | 4 | Kazakh | 335 | 201 | 87 | 149 | 8.2 | 6.1 | 2.1 |
| 16155857 | male | 45 | Kazakh | 206 | 160 | 121 | 242 | 10.8 | 5 | 5.8 |
| 16153787 | male | 46 | Han | 176 | 224 | 192 | 288 | 25.2 | 17.1 | 8.1 |
| 16160277 | male | 25 | Kazakh | 273 | 192 | 76 | 106 | 8 | 5 | 3 |
| 16160343 | male | 46 | Kazakh | 259 | 204 | 39 | 82 | 7.8 | 5.1 | 2.7 |
| 17184409 | female | 57 | Hui | 49 | 23 | 447 | 430 | 16.2 | 5.5 | 10.7 |

ALT, alanine aminotransferase; AST, aspartate aminotransferase; GGT, γ-glutamyl transpeptidase; ALKP, alkaline phosphatase; TBil, total bilirubin; DBil, direct bilirubin; IBil, indirect bilirubin.

**Supplementary Table 3.** Comparison of latency and maximal values of serum biochemical markers between 1^st^ and 2^nd^ hospitalizations of four cases.

|  | **Case #1**  **(Hepatocellular injury)** | | | **Case #2**  **(Hepatocellular injury）** | | | | **Case #3**  **(Cholestatic injury)** | | | | **Case #4**  **(Mixed injury)** | | |
| --- | --- | --- | --- | --- | --- | --- | --- | --- | --- | --- | --- | --- | --- | --- |
|  | Baseline | After treatment | Baseline | | After treatment | | Baseline | | After treatment | | Baseline | | After treatment | |
| **Latency (days)** |  |  |  | |  | |  | |  | |  | |  | |
| **1^st^ H** | 8 |  | | 9 | |  | | 12 | |  | | 8 | |  |
| **2^nd^ H** | 5 |  | | 4 | |  | | 4 | |  | | 5 | |  |
| **ALT (U/L)** |  |  |  | |  | |  | |  | |  | |  | |
| **1^st^ H** | 38 | 324 | 74 | | 243 | | 87 | | 117 | | 50 | | 205 | |
| **2^nd^ H** | 11 | 309 | 76 | | 360 | | 105 | | 228 | | 96 | | 211 | |
| **AST (U/L)** |  |  |  | |  | |  | |  | |  | |  | |
| **1^st^ H** | 32 | 134 | 65 | | 175 | | 105 | | 149 | | 20 | | 196 | |
| **2^nd^ H** | 17 | 156 | 87 | | 269 | | 66 | | 179 | | 91 | | 166 | |
| **ALP (U/L)** |  |  |  | |  | |  | |  | |  | |  | |
| **1^st^ H** | 99 | 104 | 81 | | 81 | | 175 | | 339 | | 107 | | 219 | |
| **2^nd^ H** | 89 | 110 | 95 | | 74 | | 78 | | 220 | | 92 | | 302 | |
| **TBil (μmol/L)** |  |  |  | |  | |  | |  | |  | |  | |
| **1^st^ H** | 10 | 10 | 7.6 | | 6.8 | | 22.4 | | 89.5 | | 4 | | 5.5 | |
| **2^nd^ H** | 8 | 12 | 6.8 | | 10.1 | | 19 | | 73.1 | | 6.7 | | 7 | |

H, Hospitalization; ALT, alanine aminotransferase; AST, aspartate aminotransferase; ALP, alkaline phosphatase; and TBil, total bilirubin.

**Supplementary Table 4.** The specific names of implicated DILI drugs.

| **Type** | **Specific drug names** |
| --- | --- |
| Anti-infectious agents | Doxycycline, Ceftazidime, SMZ-TMP, Levofloxacin |
| TCM^a^ | Xi-Yan-Ping, Fu-Gui-Gu-Tong, Pu-DiLan, Ku-Die-Zi, Qi-Jiao-Sheng-Bai, Shen-Mai, Yuan-Hu, Bai-Ling, Deng-Zhan-Hua, Xue-Lian, Chai-Hu, Du-Yi-Wei |
| NSALDs | Ibuprofen, Aspirin, Nimesulide |
| NM | Xiao-Chai-Hu-Tang |
| Digestive drugs | Omeprazole |
| Hormone | Dexamethasone |
| H1RAS | Promethazine |

^a^The detailed ingredient is unknown. TCM: traditional Chinese medicine. NM, natural medicine. NSALDs, nonsteroidal anti-inflammatory drugs. H1RAS, H1 receptor antagonists.

**Supplementary Table 5.** Single variable logistic regression model for risk factors of DILI among brucellosis inpatients

| **Variables** | **Odds ratio**  **(95% CI)** | **P value** |
| --- | --- | --- |
| Age, yr | 0.992 (0.977-1.007) | *0.273* |
| Gender (male vs. female) | 0.754 (0.443-1.284) | *0.298* |
| Ethnicity (non-han vs. han) | 1.120 (0.656-1.910 | *0.678* |
| Virus hepatitis (yes vs. no) | 1.420 (0.606-3.330) | *0.420* |
| Allergic history (yes vs. no) | 0.538 (0.125-2.321) | *0.406* |
| Fever, >37.2 centigrade degree (yes vs. no) | 1.670 (0.997-2.798) | 0.051 |
| Obesity^a^ (yes vs. no) | 57.915 (7.138-469.937) | ***<0.0001*** |
| Regular alcohol intake^b^ (yes vs. no) | 3.844 (1.254-11.782) | ***0.018*** |
| Eosinophilia, % | 1.057 (0.948-1.179) | *0.315* |
| Serum total protein, g/L | 1.001 (0.971-1.032) | *0.935* |
| Serum albumin, g/L | 0.933 (0.883-0.986) | ***0.014*** |
| Antibody titer of SAT, (1:100 vs. 1:50) | 1.036 (0.338-3.172) | *0.951* |
| Antibody titer of SAT, (1:200 vs. 1:50) | 1.473 (0.515-4.215) | *0.470* |
| Antibody titer of SAT, (1:400 vs.1:50) | 2.104 (0.791-4.215) | *0.136* |
| Antibody titer of SAT, (1:800 vs.1:50) | 2.900 (0.270-31.150) | *0.379* |

^a^BMI is greater than 30 for obesity. ^b^Regular alcohol intake is defined as consumption of 5 or more standard units of alcohol per day on 3 or more days during the preceding week. SAT, Standard tube agglutination test.

**1.2 Supplementary Figures**

**Supplementary Figure. 1**

**
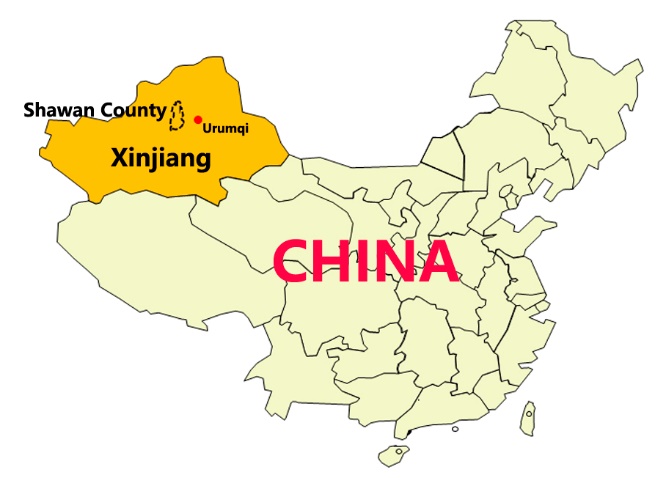
**

**Supplementary Figure. 1.** Geographic location of Shawan county in the Xinjiang autonomous region, China.

**Supplementary Figure. 2**


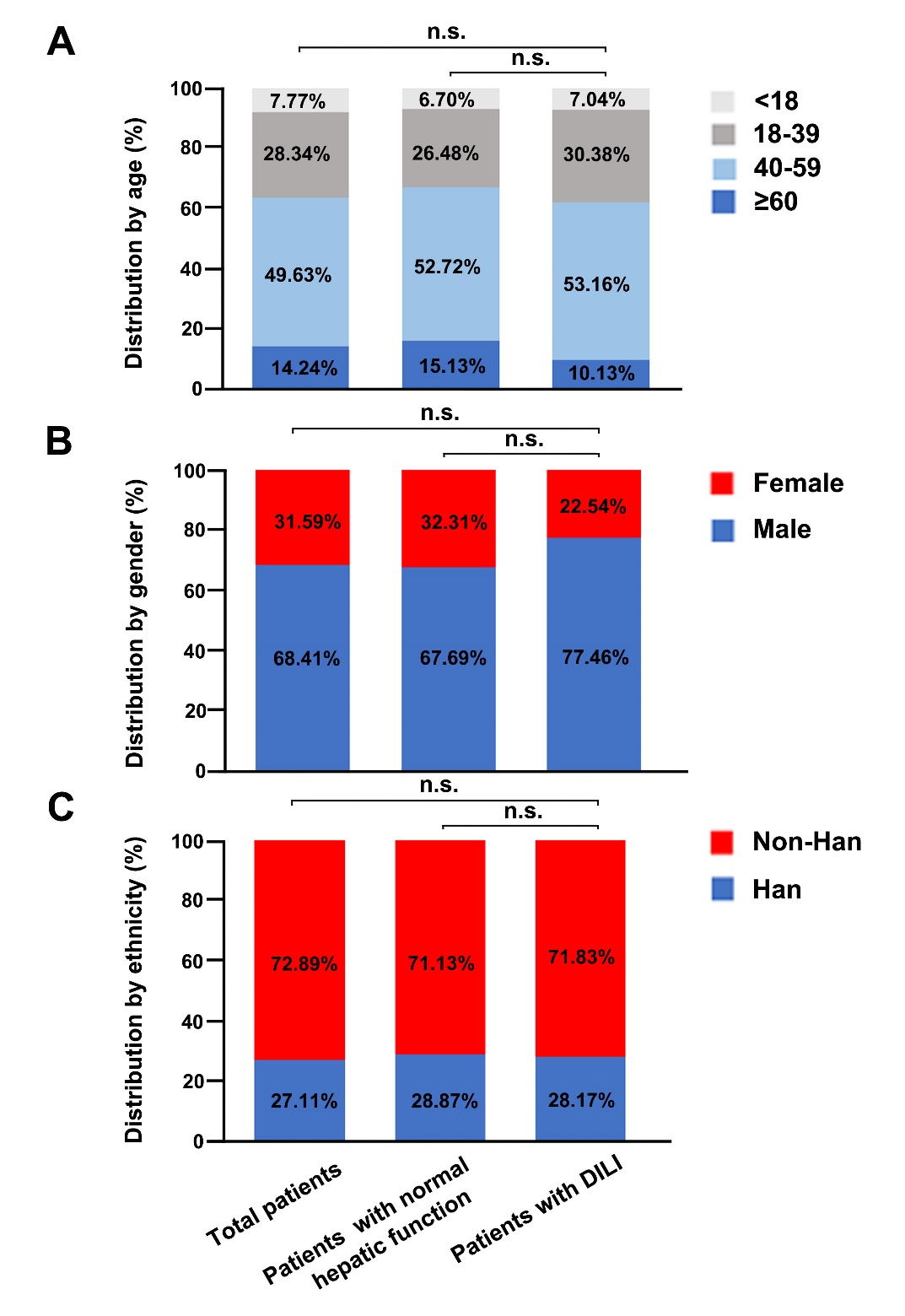


**Supplementary Figure. 2.** Age, gender and ethnic distribution among total brucellosis inpatients, brucellosis inpatients with normal liver function and brucellosis inpatients with concomitant drug-induced liver injury. The corresponding percentages are displayed on the graph. The chi-square test was used to compare differences in distribution between groups. n.s.: represents no significance.

**Supplementary Figure. 3**


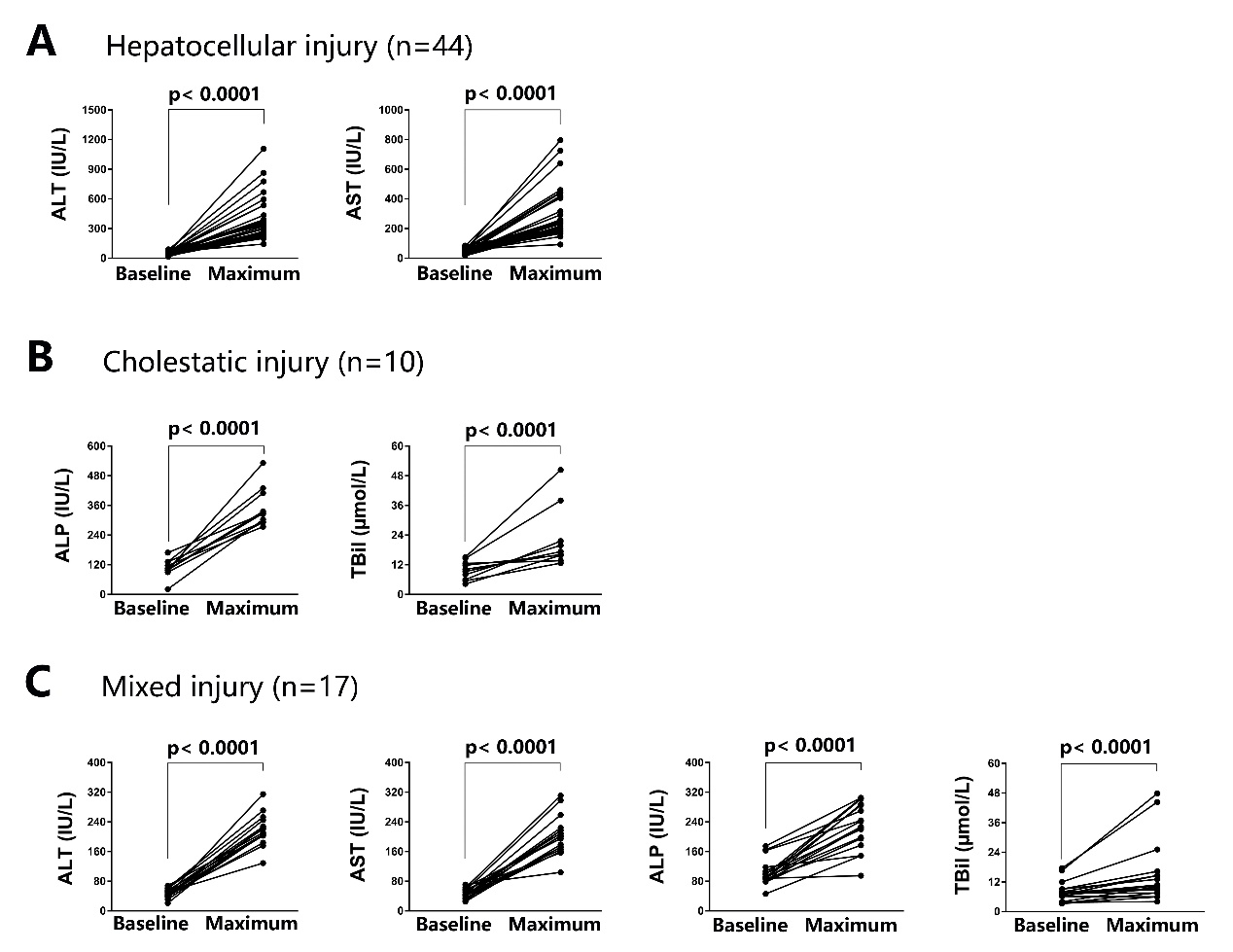


**Supplementary Figure. 3.** Comparison of the main Liver function indexes between baseline and maximal values during the course of DILI. Paired t-test was used for this analysis.

**Supplementary Figure. 4**


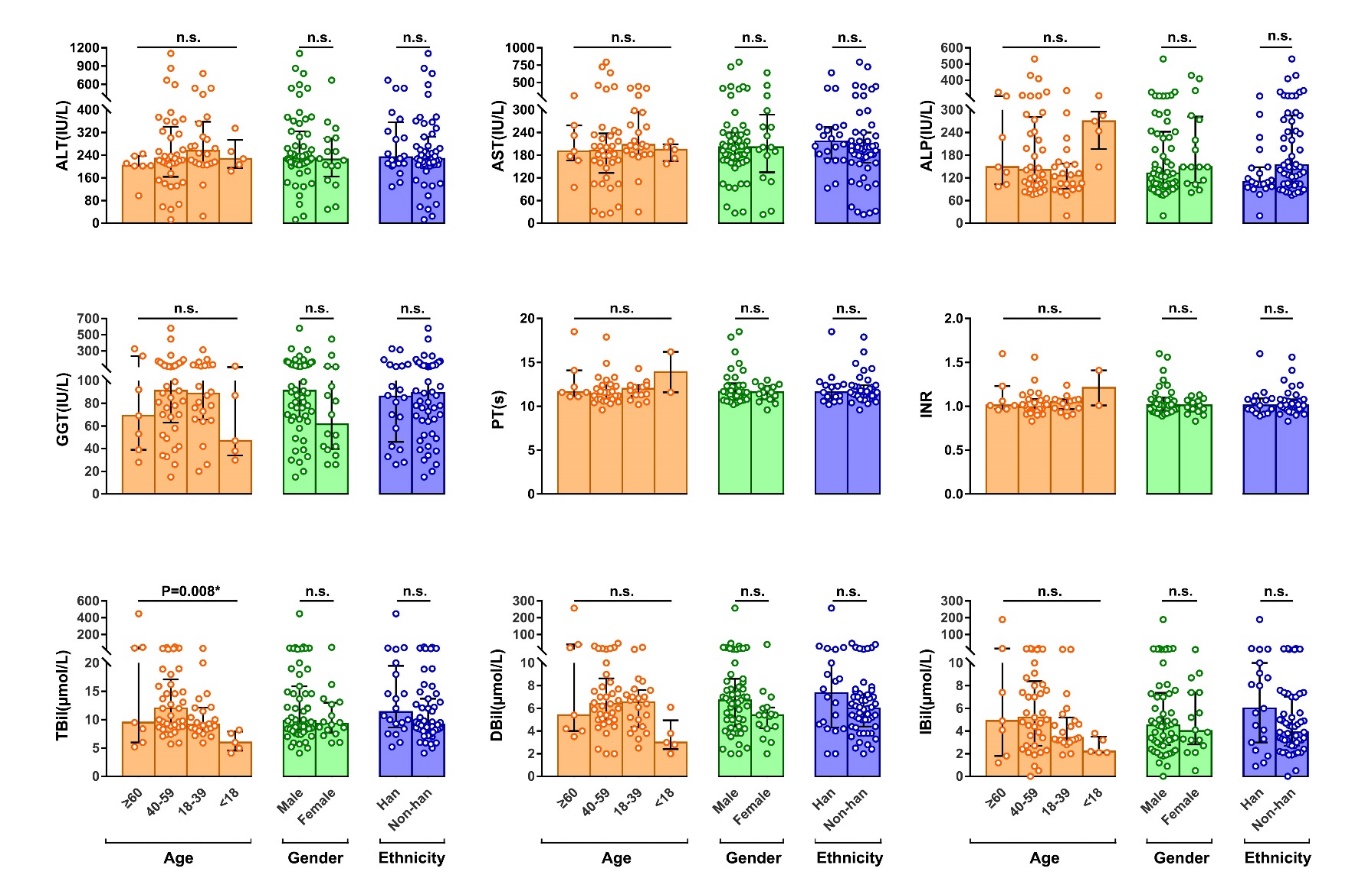


**Supplementary Figure. 4.** Comparison of maximum values of serum biochemical markers during the course of the injury among 71 cases based on gender, age, and ethnicity. All data are shown as median and interquartile range, asterisks indicate significant levels determined by either the Mann-Whitney U test or the Kruskal-Wallis test where appropriate (2-tailed; *P < .05). n.s.: not significant; ALT, alanine aminotransferase; AST, aspartate aminotransferase; ALP, alkaline phosphatase; GGT, γ-glutamyl transpeptidase; INR, international normalized ratio; PT, prothrombin time; TBil, total bilirubin; DBil, direct bilirubin; IBil, indirect bilirubin.
